# Evidence of persistent glial cell dysfunction in the anterior cingulate cortex of juvenile idiopathic arthritis children: a proton MRS study

**DOI:** 10.1186/s12969-022-00711-9

**Published:** 2022-07-27

**Authors:** Haiwei Han, Ji Hong Xiao, Yifei Weng, Hongyan Liang, Chengkun Han, Cuili Yi, Kezhao Lin, Hua Wu

**Affiliations:** 1 Department of Radiology, The first affiliated Hospital of Xiamen University, School of Medicine, Xiamen University, Xiamen, China; 2 Department of Pediatrics, The first affiliated Hospital of Xiamen University, School of Medicine, Xiamen University, Xiamen, China; 3 Department of Nuclear Medicine and Minnan PET center, The first affiliated Hospital of Xiamen University, School of Medicine, Xiamen University, 55 Zhenhai Road, Siming District, Xiamen, 361003 Fujian Province China

**Keywords:** Anterior cingulate cortex, Juvenile idiopathic arthritis, Glial cell, Proton magnetic resonance spectroscopy

## Abstract

**Background:**

This study aims to investigate whether the neurometabolites of the anterior cingulate cortex (ACC) were distinct in patients with active and inactive juvenile idiopathic arthritis (JIA) using the proton magnetic resonance spectroscopy.

**Methods:**

We measured the levels of total N-acetylaspartate (tNAA), choline (Cho), myo-inositol (ml), glutamate (Glu) and the complex of glutamate and glutamine (Glx) relative to total creatine (tCr) in ACC of each participant.

**Results:**

Compared with the healthy controls, a significant decrease of total Cho/tCr and Glx/tCr ratio in ACC occurred in active and inactive JIA group. The tCho/Cr level was negatively associated with the serum level of ESR in active JIA patients. There was no difference in NAA/tCr ratio among the three groups, which may imply that no neuron and axonal losses occurred in either active or inactive JIA patients.

**Conclusions:**

The abnormal neurometabolites in tCho/tCr and Glx/tCr in ACC may indicate that persistent dysfunction of glial cell, while neither neuron nor axonal losses occurred in active and inactive JIA patients.

**Supplementary Information:**

The online version contains supplementary material available at 10.1186/s12969-022-00711-9.

## Introduction

Juvenile idiopathic arthritis (JIA) is defined as chronic arthritis remaining unknown etiology, which begins before 16 years old and persists for a minimum of 6 weeks [1]. It comprises seven distinct categories with heterogeneous conditions defined by the International League of Associations for Rheumatology (ILAR) as follows: oligoarticular (persistent or extended), polyarticular (RF-negative or RF-positive), systemic JIA, psoriatic arthritis, and enthesitis-related arthritis (ERA) with each differing in genetic susceptibility and severity of arthritis. Any arthritis that does not fit into these categories or corresponds to >1 subtype is considered undifferentiated [2].

Empirically, a number of children and adolescents with JIA may suffer from hypersensitivity to pain, fatigue, or depression referred to as the “sick behaviors”. These changes occur may result from that inflammatory-molecular messengers of damage and infection signal the central nervous system (CNS) through afferent nerves, sentinel cells in the circumventricular organs, and transport across the blood-brain barrier (BBB) [3]. However, the exact correlations between neural activities and inflammation in chronic conditions such as JIA were scarcely explored by imaging modality, especially in the rapidly developing and perhaps vulnerable central neural system (CNS) of children and adolescents. Although previous studies have indicated that many patients with JIA can achieve inactivity or remission of the inflammation owing to the currently advanced treatment strategy (e.g., biologic agent) [4], pain and cognitive disfunction were still significant concerns for JIA patient [5]. These findings imply that centralized pain sensitization and synaptic plasticity may occur in acute and chronic inflammatory conditions [6, 7].

Localized proton magnetic resonance spectroscopy (^1^H-MRS) is a noninvasive tool for measuring neurochemical information in vivo [8]. Using MRS to explore the abnormal neurometabolites may be helpful for understanding the specific neuropathology and metabolic dysfunctions in the brain of young JIA patients. The anterior cingulate cortex (ACC) is a part of the limbic system related to the affective and attentional process of affection, cognition, as well as pain and empathy, and can be divided into three subregions, including the subgenual, rostral, and dorsal ACC [9]. These subregions interacting with multiple cortical regions usually function as relay hubs by transmitting various input signals after evaluating requirements from other regions to guide adaptive behaviors. Since some metabolite levels in the ACC are reported altered in patients suffering from chronic pain (such as osteoarthritis, chronic low back pain, and inflammatory bowel disease) [10–12], there is a high probability that the ACC is involved in young patients with JIA.

In this study, we measured the natural fluctuation in the condition of peripheral inflammation that are featured as JIA to determine how inflammation affects the neurometobolites in ACC. The JIA cohort is ideal for addressing this issue because that the condition is archetypically inflammatory and chronic, with some suffers experiencing disease flares wherein both circulating inflammatory marker and symptoms become elevated [2]. Among a variety of MRS-detectable metabolites, we focused on the levels of total N-acetylaspartate (tNAA), glutamate (Glu), the complex of glutamate and glutamine (Glx), myo-inositol (mI), and total choline (tCho) relative to total creatine (tCr) including Cr and phosphocreatine (pCr) in the ACC. This study aimed to investigate the sensitive and specific neurochemical biomarkers and neuromatabolites associating with active and inactive JIA patients. Furthermore, we explored the respective relationship of abnormal levels of neurometabolites with peripheral serum levels of pro-inflammatory cytokines and clinical assessments on patients with JIA.

## Methods

### Participants

A total of 65 JIA patients were recruited from the Pediatric Clinic of The First Affiliated Hospital of Xiamen University, China, PR. We recruited thirty-eight drug-naïve patients who is newly diagnosed as JIA according to the ILAR classification. At the same time, twenty-seven inactive JIA patients were also included in this study. In addition, we were also informed of self-report complaints, medical imaging evidence, as well as the laboratory blood tests of both JIA groups. Our sample of children and adolescents with active JIA included following subtypes: 52.6% (*n* = 20) oligoarticular, 28.9% (*n* = 11) enthesitis-related arthritis, 10.5% (*n* = 4) polyarticular, 5.2% (*n* = 2) systemic arthritis, 5% (*n* = 2) undifferentiated arthritis, while 2.6% (*n* = 1) those with obvious immune system disorders should be excluded. Twenty-seven inactive JIA patients included the following subtypes: 32.1% (*n* = 9) oligoarticular, 35.7% (*n* = 10) enthesitis-related arthritis, 22.2% (*n* = 6) polyarticular, and 7.1% (*n* = 2) undifferentiated arthritis. The patient groups were compared with 21 age- and sex-matched healthy controls (HCs, 6 females/15males, age 8.95 ± 2.01 years old (mean ± SD)). The detailed demographic information was shown in Table 1.

**Table 1:** Demographics of active and inactive JIA patients and healthy controls

	JIA-Active	JIA-Inactive	HC	***P***
	*(n* = 38)	(*n* = 27)	(*n* = 21)	
**Sex (M/F)**	27/11	21/6	15/6	*0.814*^*a*^
**Age (years)**	9.53 ± 2.19	9.81 ± 2.70	8.95 ± 2.01	*0.448*^*b*^
**SCARED**	10.34 ± 12.06	8.15± 6.60	10.38 ± 7.10	*0.621* ^*b*^
**Disease severity**				
*JADAS*	13.91 ± 6.22	0.0	-	-
*C-HAQ*	0.52 ± 0.64	0.03 (0-0.63)	-	< 0.001*^c^
*VAS*	27.10 ± 19.89	0.77 (0-20)	-	< 0.001*^c^
**Inflammation Index**				
*ESR*	28.80 ± 26.81	9.46 ± 4.67	-	< 0.005*^c^
*CRP*	9.69 ± 24.74	1.02 ± 1.94	-	0.038*^c^

^a^Chi-square test; ^b^One-way analysis of variance; ^c^Two sample t-test

^*^P < 0.05

*Abbreviations*: *JIA* Juvenile Idiopathic Arthritis, *HC* Healthy control, *M* male, *F* Female, *DSRS* The Depression Self-Rating Scale, *SCARED* The Screen for Child Anxiety Related Emotional Disorders, *JADAS*, Juvenile Arthritis Disease Activity Score, *C-HAQ* Childhood Health Assessment Questionnaire, *VAS* Visual Analogue Scale, *ESR* erythrocyte sedimentation rate, *CPR* C-reactive protein (CRP)

Further exclusive criteria were as followed: 1) either younger than eight or older than sixteen years old; 2) left-handedness; 3) brain abnormality on routine MRI scans; 4) with history of brain surgery and 5) with significant physical or psychiatric conditions; or 6) with MRI contraindications. This study was carried out according to the declaration of Helsinki and approved by the ethics committee of The First Hospital Affiliated to Xiamen University. All participants gave written Informed consent before the neurological tests and MRI scanning.

### Marker of inflammation, disease activity, and neuropsychological evaluation

Before MRI scanning, participants were assessed by a series of validated neuropsychological and clinical scales: 1) Juvenile Arthritis Disease Activity Score (JADAS-27) [13] and Childhood Health Assessment Questionnaire (C-HAQ) was chosen to assess the diseased severity and the life impact of the disability [14]. The JADAS-27 was used to evaluate the disease activity of certain JIA subgroups (oligoarticular JIA, polyarticular JIA and undifferentiated arthritis). Moreover, the Juvenile Spondyloarthritis Disease Activity Index (JSpADA) and the systemic Juvenile Arthritis Disease Activity Score (sJADAS) were used for enthesitis-related arthritis and systemic JIA, respectively [15]. Notably, the inactivity or remission of JIA was defined as JADAS ≤ 1 [15]. 2) The screen for child anxiety Related Emotional Disorders (SCARED) was selected to evaluate the emotional status of both children and adolescents with active and inactive JIA and healthy controls [16]. 3) The Visual Analogue Scale (VAS) was also selected to measure the intensity of the pain involving joint discomfort for JIA patients. Additionally, the venous blood of each JIA patient was drawn by a trained phlebotomist, and then the level of serum erythrocyte sedimentation rate (ESR) and C-reactive protein (CRP) immediately were immediately processed and measured.

### MR Scanning Acquisition

All the subjects were scanned on a 3.0-T MR scanner (Siemens Tim Verio, Erlangen, Germany), and an 8-channel phased-array coil was used to provide an optimum signal-to-noise ratio for MRI images and spectroscopy data. Sagittal T1-weighted images were acquired using 3-dimensional magnetization prepared rapid acquisitions gradient echo (MP-RAGE) sequence [2150 ms, 1100 ms, 4.38 ms, and 1 (TR, inversion time (TI), TE, and signal average)], with in-plane resolution of 256 x 256, slice thickness of 1 mm, field of view of 256 mm, and 162 slices, this provided isotropic voxel resolution of 1 mm^3^. The sagittal images were used to generate axial and coronal images using a multiplanar reconstruction (MPR) routine provided by the manufacturer. These images had the same resolution as the sagittal images. Axial T2-weighted turbo spin-echo images were acquired [8000 ms, 107, and 2 (TR, effective TE, signal averages)] with turbo factor of 15, and using 256 x 256 image matrix and slices thickness of 5 mm.

**Fig. 1:**
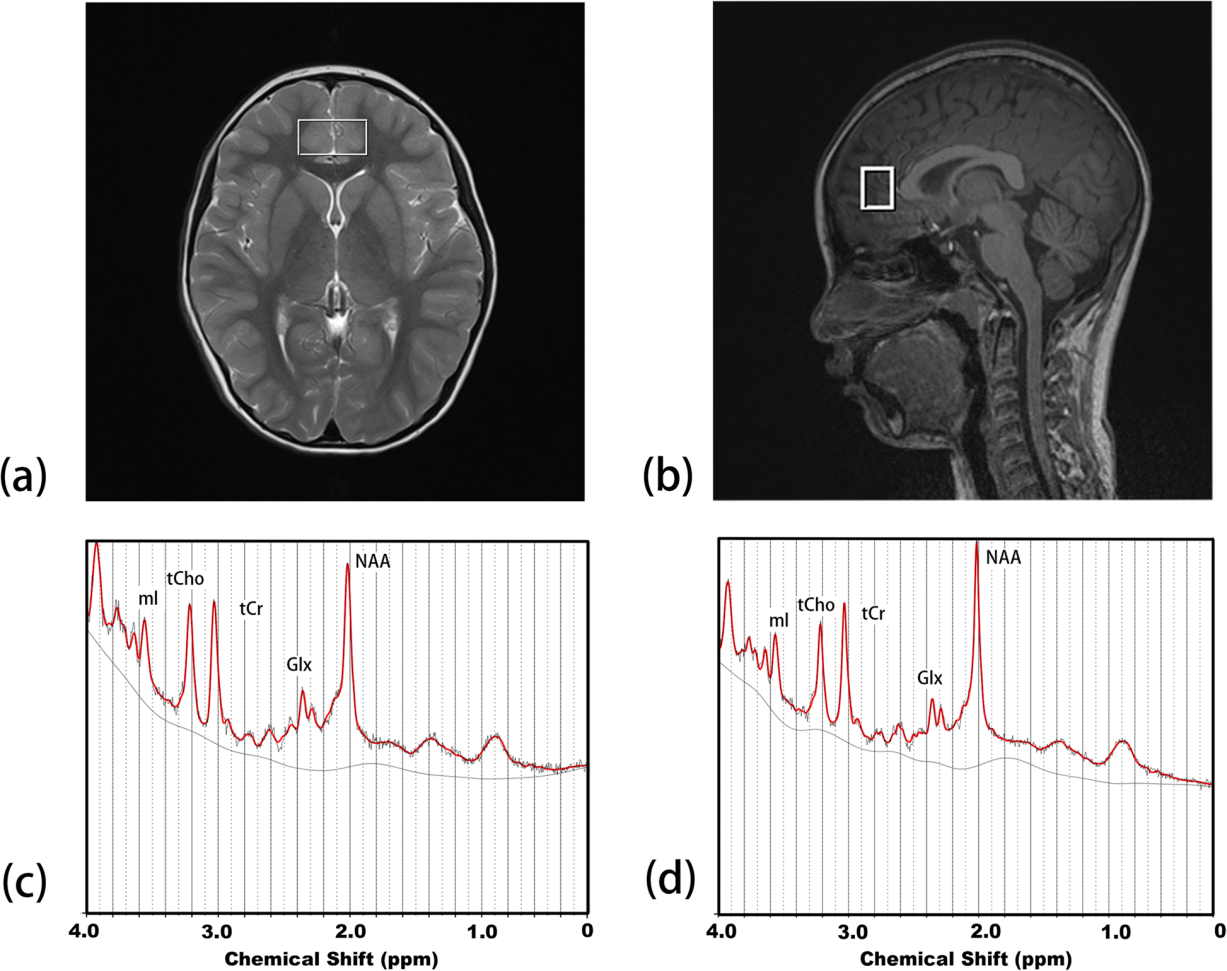
Voxel displacement. **a** Sagittal MP-RAGE T1-weighted slice and (**b**) axial T2WI showing the voxel position for the single voxel spectroscopy in the bilateral ACC (Voxel size = 30 x 15 x 20 mm3). Representative spectra acquired from the ACC in a healthy control (**c**) and a patient with active JIA (**d**)

A single voxel point RESolved Spectroscopy (PRESS) sequence with and without water suppression pulse [2000 ms, 30 ms (TR, TE)], with voxel size of 3.0 cm (left-right), 2.0 cm (anterior-posterior), 1.0 cm (head-foot), 512 sampling points, and 2 kHz bandwidth, was used to acquire data from the ACC (Fig. 1) and for water-unsuppressed data, 5 signal averages were acquired [17].

### ^1^H-MRS Data processing

Spectral analysis was performed by using the linear combination model (LC model, version. 6.3-1J) software, a user-independent time-domain fitting routine that uses a basis set of concentration-calibrated model spectra of individual metabolites to estimate the relative concentrations of similar brain metabolites from in vivo spectral data. The metabolite content was normalized to that of tCr levels. As commanded by the LC model software, concentrations and ratios with Cramer-Rao lower bounds (CRLB) of more than 20% were not used for analysis. Of note, glutamine resonance was fit poorly with CRLB (> 20%) and hence were not used in the analysis. The final analysis included only those metabolites with a Cramer-Rao lower bound (CRLB) <20% [18].

### Statistical analysis

Statistical analyses were performed using SPSS statistics 21.0 (SPSS, Chicago, IL). The sex difference between the groups was tested by Chi-Square analysis. The Kolmogorov-Smirnov test was applied to measure the normality of the quantitative data. Subsequently, the group differences in normally distributed data were assessed using a two-tailed student’s t-test; whereas a non-parametric test, the Mann-Whitney U test, was used for data that were not normally distributed. The group differences of the levels of neurometabolites among the three groups were analyzed using both parametric test (analysis of variance) and non-parametric test (Kruskal-Walls H-test). The Spearman correlation analysis was conducted between every two indices, which were derived from the significant difference of the neurometabolites, inflammation factors, clinical measurements, and psychological test score. The *P values < 0.05* were considered statistically significant.

## Results

### Demographics

A total of sixty-five active and inactive JIA and twenty-one healthy control subjects completed the data collection. The demographic and clinical characteristics of the subjects are present in Table 1. Age, gender ratios were not significantly different between the two groups. The JADAS-27 scores (sJADAS and JSpADA for sJIA and ERA) for active and inactive JIA patients were 13.91 ± 6.22 (Mean ± SD) and 0, respectively. The C-HAQ and VAS scores of active JIA patients were significantly higher than that of inactive JIA patients (*p <0.001*).

**Fig. 2:**
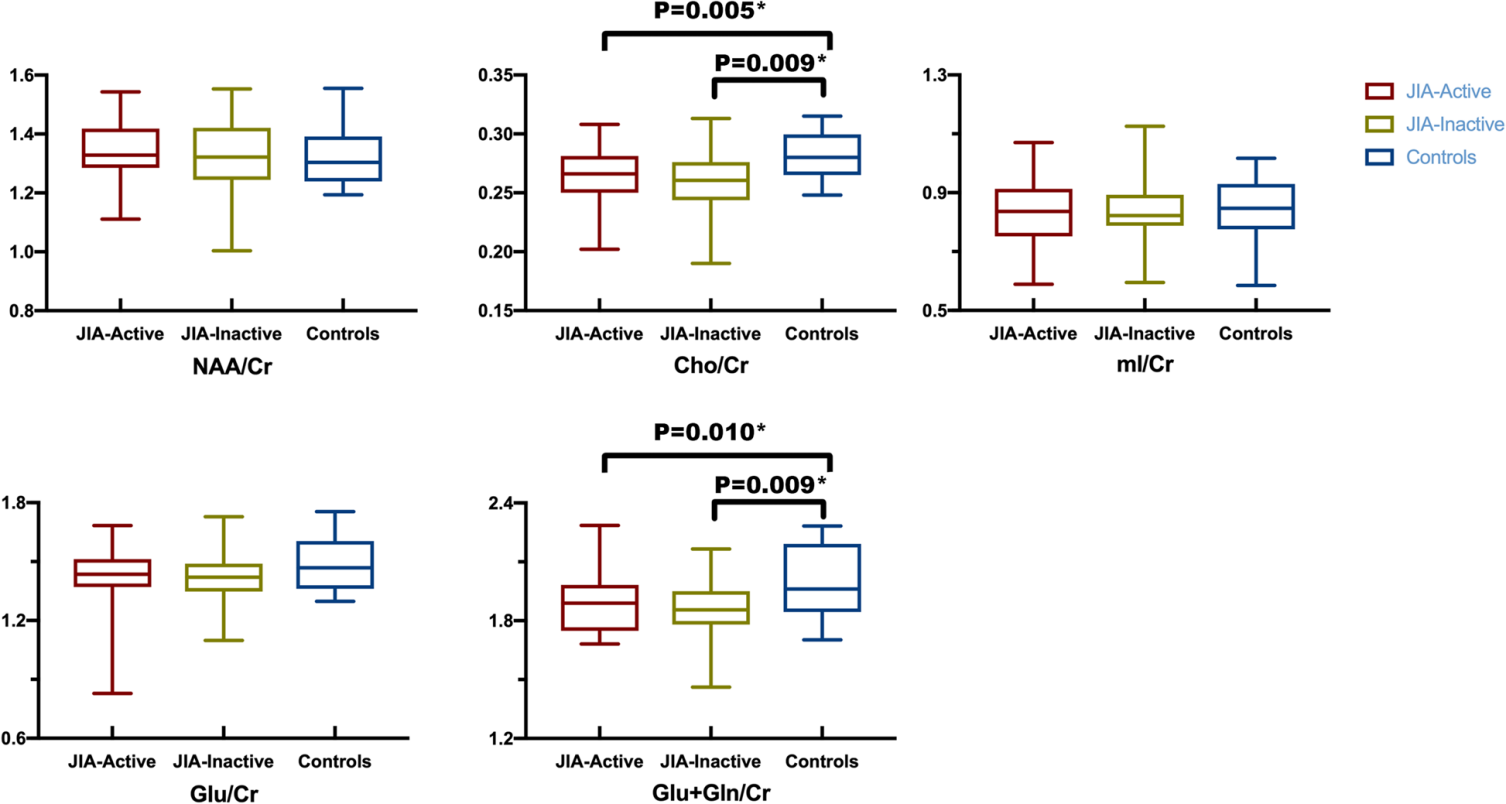
The mean ratio of brain metabolites of three groups (JIA-active, JIA-inactive, and healthy control). The JIA-active and inactive groups have lower levels of tCho/Cr and Glx/tCr than healthy control group

The levels of NAA/tCr, tCho/tCr, mI/tCr, Glu/tCr, Glx/tCr in active and inactive JIA patients and healthy controls were listed in Table 2 and Fig. 2. The level of tCho/tCr and Glx/tCr in active and inactive JIA patients were significantly lower than those in health controls (*p <0.05*). The NAA/tCr, Glu/tCr, and mI/tCr levels did not significantly differ from active JIA, inactive JIA and healthy controls. Neither tCho/tCr nor Glx/tCr levels for active and inactive JIA patients showed no significant difference. We also reported the non-parametric test p-levels in Supplementary Table 1.

**Table 2:** Comparison of neurometabolites in anterior cingulate cortex between active and inactive JIA patients and healthy controls

	JIA-Active	JIA-Inactive	HC	***F***	***P***
**NAA/Cr**	1.34 ± 0.09	1.32 ± 0.13	1.32 ± 0.09	*0.247*	*0.781* ^a^
**Cho/Cr**	0.26 ± 0.02	0.26 ± 0.03	0.28 ± 0.02	*4.867*	***0.010*** ^a^*
**ml/Cr**	0.83 ± 0.11	0.83 ± 0.10	0.85 ± 0.11	*0.283*	*0.754* ^a^
**Glu/Cr**	1.44 ± 0.15	1.42 ± 0.14	1.49 ± 0.14	*1.287*	0.282^a^
**Glu+Gln/Cr**	1.89 ± 0.15	1.87 ± 0.16	2.00 ± 0.18	*4.413*	*0.015* ^a^*

^a^ One-way analysis of variance

^*^P < 0.05

*Abbreviations*: *JIA* Juvenile Idiopathic Arthritis, *HC* Healthy control

**Fig. 3:**
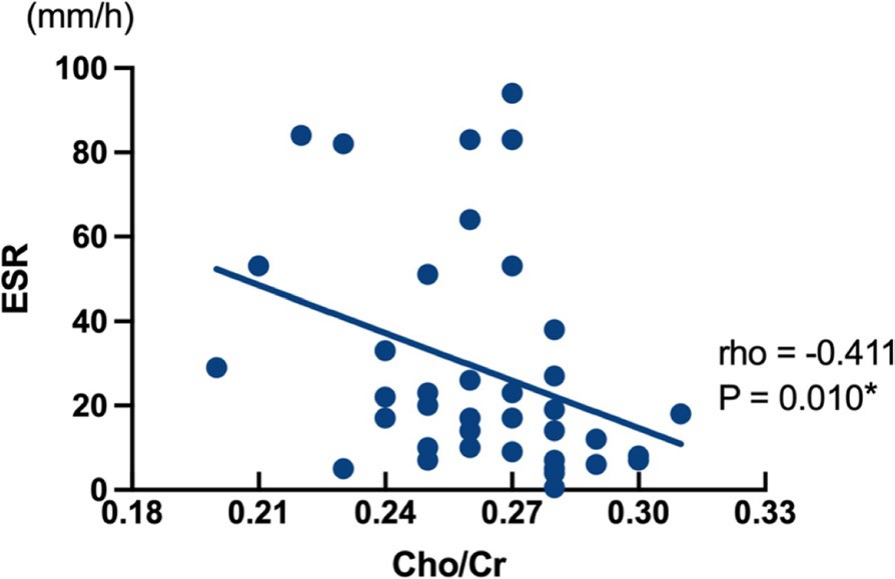
The correlation analysis. The tCho/tCr level demonstrated an inversed correlation with ESR in acitive JIA patients

There were negative correlations between tCho/tCr and peripheral levels of ESR in active JIA patients (*r = -0.411, p = 0.010*) (Fig. 3). There was no correlation between tCho/tCr levels and peripheral levels of ESR for inactive JIA patients. No correlation was found between Glx/tCr and peripheral serum levels of ESR and CRP in active and inactive JIA patients. JADAS (sJADAS and JSpADA for sJIA and ERA) and VAS scores for active and inactive JIA patients were not correlated with tCho/tCr (*r = 0.051, p = 0.761; r = 0.103, p = 0.540, respectively)* and Glx/tCr levels (*r = -0.038, p = 0.821; r = -0.212, p = 0.202, respectively*) in ACC.

## Discussion

This study revealed the abnormality of neurometabolites in JIA patients, which may indirectly reflect the vulnerability of microstructural in the condition of chronic inflammation. Firstly, we observed the abnormality in tCho/tCr and Glx/tCr of ACC indicating the dysfunction of glial cells in both active and inactive JIA patients. whereas no significant difference was found between the two patient groups. Furthermore, the levels of tCho/tCr were negatively associated with peripheral levels of ESR in active JIA patients. Secondly, there was no significant difference in the level of NAA/tCr when respectively compared the active and inactive JIA patients with healthy controls, which might imply that no neuron and axonal losses occurred in either active or inactive JIA patients. To the best of our knowledge, this is the first study to demonstrate a decrease of tCho/tCr and Glx/tCr ratios in ACC of JIA patients by using ^1^H-MRS.

Our findings of the decreased tCho/tCr ratio in ACC of both active and inactive JIA indicated the occurrence of biochemical perturbation such as a decelerated turnover of the membrane. The Cho is a marker to detect the turnover of glial cell membrane in CNS and is also an essential precursor of acetylcholine and membrane lipids, phosphatidylcholine, and sphingomyelin [19–22]. The reduction of tCho/tCr levels in the ACC of JIA was consistent with previously reported alterations in the brain phospholipid metabolism, which may lead to altered lipid membrane turnover and neuroplasticity [23, 24]. The decreased tCho/tCr ratio also supported the impaired astrocytic functioning and altered neuroplasticity in JIA patients. Furthermore, increases in ESR and CRP together with other clinical assessments such as swollen joints could represent an important metric for measuring disease severity and benchmarks for the success of treatment [25–27]. In our study, the tCho/Cr level was negatively associated with peripheral levels of ESR, indicating that dysfunction of glial cells especially astrocyte cells occurred during acute inflammation in JIA. The tCho/Cr level showed no significant difference between active and inactive JIA patients, implying that dysfunction of glial cells in active inflammatory conditions may not be reversible, and glial cells may be vulnerable to insult of inflammatory damage during the developing CNS for children and adolescents, and this can persist in the inactive phase of JIA. These findings may explain why many inactive JIA patients have persistent pain and fatigue symptoms. It should be noted that the result of decreased tCho/tCr levels in JIA was not consistent with the previous studies on adult rheumatic autoimmune diseases such as rheumatoid arthritis, systemic lupus erythematosus and systemic sclerosis [8]. The increase of Cho/Cr ratio was usually observed in different adult rheumatic autoimmune diseases, implying that glial reactivity occurred in response to inflammation and involvement of cerebral myelin [8]. The period of childhood and adolescence is considered as an important and yet vulnerable course of immense development that is distinct from adulthood, and therefore should not be directly compared with.

In current study, our main observations on glutamatergic neurometabolites are threefold: (1) no significant difference was found in Glu/tCr ratio level in ACC between active and inactive JIA patients and controls; (2) Glx/tCr ratio level was lower within the ACC in active and inactive JIA patients compared with controls, and no significant difference showed in active and inactive JIA patients; (3) no relationships were found between the Glx /tCr level of ACC any clinical variables in JIA patients. The glial cells such as astrocytes are key regulators of glutamine-glutamate homeostasis. A growing body of evidence shows impaired astrocytic functioning and abnormal glutamate/glutamine homeostasis in a series of CNS diseases such as major mood disorders and neurodegenerative disorders [28–30]. Recent evidence indicates that inflammatory mediators might regulate extracellular glutamatergic concentrations by exerting a profound effect on the functioning of glial cells including astrocytes, oligodendrocytes, and microglia that regulate glutamatergic levels under both physiological and pathological conditions [31]. The progression of glutamatergic dysfunction in chronic inflammation may result in atrophic changes of astrocytic and oligodendrocyte cells [28]. The decreased Glx/tCr ratio in our study may also indicate that dysfunction of glial cells in ACC for JIA patients.

Compared with healthy controls, we failed to detect the differences in the levels of NAA between JIA patients and healthy cohort. The NAA is synthesized in the mitochondria of neurons, then transported into neuronal cytoplasm and along axons [32]. The NAA is exclusively found in the peripheral and central nervous system and is detected in both gray matter and white matter. The rates of NAA synthesis provide an indication of the rate of glucose consumption by neurons, containing information on the level of neuron activity [8]. The NAA peaks were used as a marker of density, functionality, and neuronal integrity, and a decrease in concentration may reflect the loss of neurons or neuronal dysfunction. Accordingly, this negative finding was in good agreement with that no neuronal and axonal losses occurred in JIA patients.

Moreover, we also did not find any aberrances of ml in JIA. The mI is one of the most abundant metabolites in the brain, with the peak at 3.56 ppm on short TE ^1^H-MRS [33]. It is a sugar-like molecule that acts as an osmolyte and is involved in the various key biochemical process, such as signal transduction, phosphorylation of target protein, chromatin remodeling, and gene expression [33, 34]. The mI can be found mainly within astrocytes and cannot cross the blood-brain barrier, and the increase of mI/Cr level is thought to reflect glial proliferation or an increase in glial cell size [35]. The stable ml level in ACC might indicated that no gliosis and the increasement of glial cell size occurred in JIA.

This study has some limitations. Firstly, the sample in this study was relatively small. JIA unifies all forms of chronic childhood arthritis, and not all subtypes of JIA were included in this study. Secondly, we performed a cross-sectional study and only analyzed a single area of the brain, while other brain regions such as the insula and putamen may be included in the future. To address above-mentioned disadvantages, the further studies with a larger sample size and longitudinal follow-up are needed before a definitive conclusion can be drawn. Thirdly, although ESR and CRP are metrics of inflammatory activity, they could not show the same level of mechanistic specificity as pro-inflammatory cytokines, such as IL-6, TNF-α, and IL-1β, which should be also measured in the future work. Last but not the least, the sex effect should be also considered in the future study as there is evidence for sexual-specific differences in acute inflammatory paradigms.

## Conclusion

In conclusion, our study revealed the abnormal neurometabolites including tCho/tCr and Glx/tCr of ACC in JIA patients, which indicated the persistent dysfunction of glial cell. However, no neuron and axonal losses occurred in active and inactive JIA patients.

## Supplementary Information

**Additional file 1.**

## Data Availability

The original contributions presented in the study are included in the article, further inquiries can be directed to the corresponding authors.
