# Supplementary material for: Evidence of persistent glial cell dysfunction in the anterior cingulate cortex of juvenile idiopathic arthritis children: a proton MRS study
Source: Pediatr Rheumatol Online J. 2022 Jul 27;20:53. doi: 10.1186/s12969-022-00711-9 (PMC9327147; doi:10.1186/s12969-022-00711-9)
Supplement: Supplementary file 1 — Additional file 1. [file 12969_2022_711_MOESM1_ESM.docx]

**Supplementary Table 1.** **Kruskal-Walls H-test on levels of metabolites**

|  | **JIA-Active** | **JIA-Inactive** | **HC** | **H** | **P** |
| --- | --- | --- | --- | --- | --- |
| **NAA/Cr** | 1.34 ± 0.09 | 1.32 ± 0.13 | 1.32 ± 0.09 | 0.849 | 0.654 |
| **Cho/Cr** | 0.26 ± 0.02 | 0.26 ± 0.03 | 0.28 ± 0.02 | 8.153 | 0.017* |
| **ml/Cr** | 0.83 ± 0.11 | 0.83 ± 0.10 | 0.85 ± 0.11 | 1.128 | 0.569 |
| **Glu/Cr** | 1.44 ± 0.15 | 1.42 ± 0.14 | 1.49 ± 0.14 | 1.887 | 0.389 |
| **Glu+Gln/Cr** | 1.89 ± 0.15 | 1.87 ± 0.16 | 2.00 ± 0.18 | 46.524 | 0.038* |
